# The MUnich SArcopenia Registry (MUSAR): Paving the way for increased visibility, more frequent diagnoses and innovative new treatment

**DOI:** 10.1007/s00391-025-02428-2

**Published:** 2025-04-08

**Authors:** Olivia Tausendfreund, Uta Ferrari, Christopher Held, Sebastian Martini, Katharina Mueller, Hannah Reif, Michaela Rippl, Sabine Schluessel, Ralf Schmidmaier, Michael Drey

**Affiliations:** Department of Medicine IV, LMU University Hospital, LMU Munich, 80336 Munich, Germany

**Keywords:** Muscle wasting, Clinical trial, Disease-specific registry, Osteoporosis, Osteosarcopenia, Muskelschwund, Klinische Studie, Krankheitsspezifisches Register, Osteoporose, Osteosarkopenie

## Abstract

**Background:**

The consensus definition of sarcopenia enables a clear diagnostic algorithm. The syndrome can now also be coded in Germany (International Classification of Diseases 10, ICD-10 GM 62.50). Compared to the estimated prevalence it is still significantly underdiagnosed. Current treatment options include resistance training and a protein-rich diet, while pharmacological options are still missing.

**Objective:**

The Munich Sarcopenia Registry (MUSAR) aims to raise awareness of the syndrome and affected individuals. Additionally, it seeks to gain insights into risk factors, causes and treatment approaches. This publication conducts an initial analysis of 90 patient datasets with varying degrees of sarcopenia and examines the cohort for key geriatric parameters.

**Material and methods:**

Since 2018 patients from the geriatric clinic of the Ludwig Maximilians University Munich have been able to contribute their data to the registry. Sociodemographic, anthropometric, functional, and laboratory data are collected in a web-based registry.

**Results:**

Compared to patients without sarcopenia, patients with sarcopenia are significantly older, have more comorbidities and show poorer functional performance as well as reduced quality of life.

**Discussion:**

The results highlight the urgent need for further research and the development of new forms of treatment to improve the quality of life and independence of these patients. Challenges such as difficult recruitment complicate this endeavor. The MUSAR aims to minimize these issues and provides a valuable basis for generating extensive data through the systematic collection of patient data during hospital stays.

**Supplementary Information:**

The online version of this article (10.1007/s00391-025-02428-2) contains supplementary material, which is available to authorized users.

## Abstract (de)

**Hintergrund:**

Die Konsensusdefinition der Sarkopenie ermöglicht einen klaren diagnostischen Algorithmus. Das Syndrom kann in Deutschland auch kodiert werden (ICD-GM 62.50). Im Vergleich zur geschätzten Prävalenz ist es jedoch deutlich unterdiagnostiziert. Behandlungsoptionen sind Krafttraining und proteinreiche Ernährung, während medikamentöse Therapien fehlen.

**Ziel:**

Das Münchner Sarkopenie Register (MUSAR) soll helfen, das Bewusstsein für das Syndrom und die Betroffenen zu steigern. Zudem sollen Erkenntnisse zu Risikofaktoren, Ursachen sowie therapeutische Ansätze gewonnen werden. Diese Publikation liefert eine erste Auswertung der Daten von 90 Patienten verschiedener Sarkopenieschweregrade und untersucht diese hinsichtlich wichtiger geriatrischer Parameter.

**Materialien und Methoden:**

Seit 2018 haben Patienten der geriatrischen Klinik der Ludwig-Maximilians-Universität München, die Möglichkeit, ihre Daten dem Register zur Verfügung zu stellen. Soziodemografische, anthropometrische, funktionelle und laborchemische Daten werden in einem webbasierten Register erfasst.

**Ergebnisse:**

Im Vergleich zu nichtsarkopenen Patienten sind sarkopene Patienten signifikant älter, haben mehr Komorbiditäten und weisen eine schlechtere funktionelle Leistungsfähigkeit sowie Lebensqualität auf.

**Diskussion:**

Die Ergebnisse verdeutlichen den dringenden Bedarf an weiterer Forschung und Entwicklung neuer Therapien, um die Lebensqualität und Selbstständigkeit dieser Patienten zu verbessern. Herausforderungen wie die schwierige Rekrutierung erschweren dieses Vorhaben. MUSAR versucht, diese Hürden zu minimieren und bietet durch systematische Datenerfassung während der Krankenhausaufenthalte eine wertvolle Grundlage für eine umfangreiche Datenbank.

In order to improve functional ability as one of the main goals of the World Health Organization baseline report “Decade of healthy ageing” the diagnosis and treatment of sarcopenia play an important role [1]. Sarcopenia is defined as progressive and generalized loss of muscle mass with a simultaneous reduction in muscle strength and/or function [2]. Sarcopenia not only poses a risk for loss of activities of daily living, negatively impacting the quality of life but is also associated with a high risk of falls, fractures, hospitalization and mortality [2].

Despite its presumably high prevalence, awareness of sarcopenia remains low among healthcare professionals. A survey of US physicians revealed that less than 20% of internists and family medicine physicians are familiar with the term sarcopenia, whereas geriatricians (70%) and physical medicine and rehabilitation physicians (41%) showed higher familiarity [3]. A survey of healthcare professionals in the Netherlands revealed that two thirds knew the concept of sarcopenia but only one in five knew how to diagnose it [4]. In recent years, diagnostic algorithms for sarcopenia have been established through collaborations between working groups in Europe, Asia and the USA [5–7]. The next steps from the newly formed Global Leadership Initiative on Sarcopenia include the development of an international consensus on the definition and diagnosis [7]. In the revised consensus definition of the European Working Group on Sarcopenia in Older People (EWGSOP2) from October 2018, muscle strength by hand grip strength and physical performance by chair rising time have become the main diagnostic elements, with the SARC‑F (Strength, Assistance with walking, Rise from a chair, Climb stairs and Falls) questionnaire for screening in advance [7, 8]. In Germany, sarcopenia can be coded (International Classification of Diseases 10, ICD-10-GM M62.50) since January 2018 [9].

The exact pathophysiology behind the syndrome remains unclear. Due to this knowledge gap, a specific pharmacotherapy for sarcopenia is still not available. The only effective treatment options are resistance training and a protein-rich diet [10, 11]. Particularly concerning secondary sarcopenia, which unlike primary sarcopenia is not solely age-related and may be influenced by other comorbidities, further insights into treatment approaches would be very valuable.

In response, we have initiated the Munich Sarcopenia Registry (MUSAR) and now present initial findings of geriatric patients either at risk of sarcopenia or diagnosed with probable sarcopenia or sarcopenia.

## Construction and content

### Aim of the registry and first data

The MUSAR aims to enhance awareness of sarcopenia. In the long term we seek to establish a database for studying modifiable risk factors and the underlying pathophysiology of sarcopenia. The first data shown here exemplarily investigate whether there are significant differences in comorbidities, functional impairments or quality of life among patients with different stages of sarcopenia or those at risk of developing it.

### Design of the registry

The sarcopenia registry (Project title: Identification of ICD-based SARcopenia, I(C)DSAR, The MUnich SArcopenia Registry (MUSAR) ethical vote no. 17-874) was founded in July 2018 at the Department of Medicine IV, LMU University Hospital Munich, Germany under consideration of the European General Data Protection Regulation (EuGDPR), enforceable beginning 25 May 2018.

**Fig. 1:**
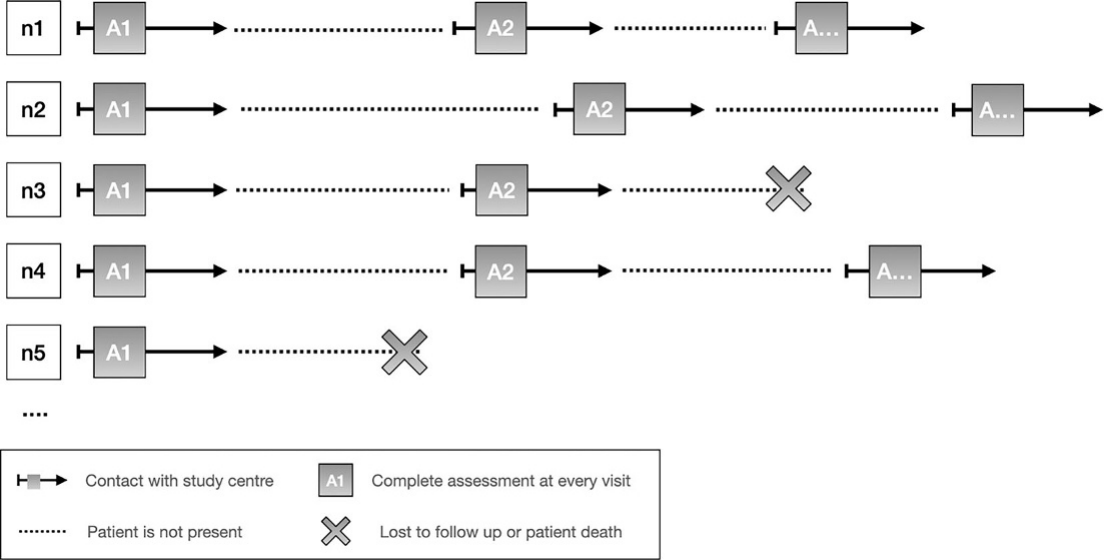
Individual assessment timelines exemplified by 5 different courses (n1-5)

In our case, patients are recruited during their hospital stay. This results in different time intervals between assessments. Figure 1 shows an overview of possible assessment timelines for different participants.

### Database and quality control

A web-based open-source database with expandable modules for deep phenotyping was established using LibreClinica by ReliaTec© (ReliaTec GmbH, Munich, Germany) and was validated for further clinical trials.

Data quality parameters will be continuously assessed and quantified. This quality control can be ensured through a sampling framework with predefined study variables, documentation of the frequency and distribution of missing data as well as checking for range value violations and follows existing frameworks and data quality guidelines [12].

### Characteristics of participants

From July 2018 onwards patients with sarcopenia or with a risk of sarcopenia have been recruited from the outpatient and inpatient facilities of the geriatric department of the Ludwig Maximilians University (LMU) Hospital in Munich, Germany. There are no exclusion criteria regarding primary or secondary sarcopenia or whether patients are already undergoing treatment. The only requirement is that patients must be at least 65 years old and capable of giving informed consent.

### Measurements and assessments in the registry

To help establish a global and comprehensive understanding of the risk factors, causes and impacts of sarcopenia on patients’ lives, we conduct a complete assessment at every contact with the study center. A detailed list of the collected data, the conducted tests and questionnaires can be found online in the *Appendix*

### First data and statistics

To provide initial insights into the cohort that we will continue to investigate in the future, we analyzed 30 complete patient data sets for each syndrome stage. This includes patients identified as at risk of sarcopenia, defined by a SARC‑F score of 4 or higher and those with probable sarcopenia, characterized by normal muscle mass but impaired muscle function (defined as handgrip strength below 16 kg for women and below 27 kg for men, and/or a chair rising test time exceeding 15 s for both sexes) and patients diagnosed with sarcopenia, marked by reduced muscle mass (with cut-off thresholds for skeletal muscle mass indexes of less than 5.5 kg/m^2^ for women and less than 7.0 kg/m^2^ for men) in addition to impaired muscle function [8]. Patients were subsequently divided into subgroups. In total data from 90 patients collected between July 2018 and August 2024 were analyzed using ANOVA and χ^2^-tests (IBM SPSS v.29.0, IBM Corp, Armonk, NY, USA).

## Results

Table 1 presents the baseline data of 30 patients in each subgroup: those identified as at risk of developing sarcopenia, those diagnosed with probable sarcopenia and those diagnosed with sarcopenia.

**Table 1:** Characteristics of patients from MUSAR (Munich Sarcopenia Registry)

	All (*n* = 90)	At risk (*n* = 30)	Probable sarcopenia (*n* = 30)	Sarcopenia (*n* = 30)	*p*-value
Age (years)	83 (7)	80 (7)	84 (7)	85 (6)	**0.003**
Outpatient (*n*) (%)	55 (61)	23 (76)	18 (60)	14 (47)	0.089
Female patients (*n*) (%)	67 (74)	26 (86)	23 (77)	18 (60)	0.057
Number of medications	9 (3)	8 (3)	9 (4)	10 (3)	0.155
Body mass index (kg/m^2^)	25.1 (5.5)	26.5 (5.7)	27.4 (5.4)	21.6 (3.2)	**<0.001**
Charlson comorbidity index (points)	3 (2)	1 (1)	2 (2)	3 (2)	**<0.001**
Mini nutritional assessment short form (points)	10 (3)	11 (3)	10 (3)	9 (3)	**0.001**
Mini mental state examination (points)	27 (4)	28 (2)	27 (4)	26 (4)	0.096
Activity of daily living (points)	79 (25)	93 (11)	73 (24)	68 (30)	**<0.001**
Short physical performance battery (points)	6 (3)	9 (3)	4 (3)	4 (2)	**<0.001**
Sarcopenia quality of life questionnaire (points)	52 (15)	56 (15)	49 (14)	52 (14)	**0.049**

All measures are presented as mean and standard deviation (SD) unless otherwise noted, bold *p*-values are below 0.05. Charlson Comorbidity Index (CCI, 0–37 points), Mini Nutritional Assessment Short Form (MNA-SF, 0–14 points), Mini Mental State Examination (MMSE, 0–30 points), Activity of Daily Living (ADL, 0–6 points), Short Physical Performance Battery (SPPB, 0–12 points), Sarcopenia Quality of Life Questionnaire (SarQoL, 0–100 points). A detailed explanation of the conducted tests, questionnaires and scales can be found online in the *Appendix*.

## Utility and discussion

Our results show that sarcopenic patients are significantly older, have more comorbidities and reduced functional capacity and have lower quality of life compared to non-sarcopenic patients. As indicated by the low Activity of daily living (ADL) scores, they exhibit a higher level of dependence on caregivers. Furthermore, both nutrition and Body mass index (BMI) appear to be associated with sarcopenia or the development of sarcopenia.

Additionally, there is a noticeable trend toward poorer cognitive function among sarcopenic patients, although not statistically significant. The findings from our initial cohort of 90 patients align with previously published results [2]. Many of these parameters worsen as probable sarcopenia progresses to manifest sarcopenia, emphasizing the importance of early detection and intervention to prevent further muscle wasting. In the next section, we therefore outline the need for further scientific approaches and the specific advantages and disadvantages that registry-based methods such as the MUSAR can offer.

### Sarcopenia research and awareness: urgently needed

Lewis et al. labelled the rise of age-associated musculoskeletal syndromes and diseases, due to higher life expectancy, change in lifestyle and insufficient interventions as a main burden of the twenty-first century healthcare system. They also note that other medical conditions are commonly aggravated, and psychological distress is worsened by musculoskeletal disorders [13]. The broad negative impact of sarcopenia, beyond its purely functional aspects, is also evident in our sample (Table 1).

In addition to the high burden imposed by the syndrome itself, the significant knowledge gap further complicates patient care. The lack of awareness among practicing physicians, as highlighted in the introduction, underlines the need to raise awareness and build expertise. A reason for this gap could be the lack of consensus on diagnostic criteria, which has also made the design and implementation of treatment trials on sarcopenia very challenging in the past [11, 14].

### Underrepresentation of cohorts of older adults in research

It is imperative to state that the cohort of older adults is underrepresented in clinical research and trials [15]. This gap in representation becomes even more consequential when one considers the development of evidence-based medical guidelines intended to guide treatment decisions for a broad patient spectrum. Paradoxically, these guidelines frequently omit adequate representation of older individuals. Consequently, older adult patients may not experience the intended benefits of treatment, or worse, could face unnecessary harm [15]. Challenges in research on geriatric patients often arise from recruitment issues, such as cognitive impairment and comorbidities. Maintaining follow-up with older patients presents its own set of difficulties. Many may encounter transportation barriers or experience fluctuations in their health.

### Registry-based research

Every registry design follows its purpose. Administrative registries collect the least amount of data and are predominantly used to measure the prevalence of certain diseases or syndromes [16].

Clinical registries aim to not only identify patients but also to learn as much as possible about them, their disease, progression, intervention and outcomes [16]. Gliklich et al. differentiated clinical registries further, between product registries, where patients are exposed to a specific medication or medical product and outcomes and safety are examined; next to this health service registries, where patients undergo a particular procedure and lastly, disease or condition registries, where the presence of a specific condition serves as an inclusion criterion [17].

The MUSAR fits the latter category and therefore offers a much broader perspective on patients and syndromes compared to conventional predefined study protocols. As patients are recruited from the acute geriatric ward and geriatric day clinic, the study cohort can be considered representative of a wide spectrum of geriatric treatment settings. Moreover, the cohort is even more representative as it includes not only patients suffering from sarcopenia but also those classified as at risk of sarcopenia through the SARC‑F assessment or classified as having probable sarcopenia. This can help identify early risk factors for the development or aggravation of sarcopenia. In this context longitudinal observations will help to identify new prognostic and modifiable factors, including harmful and beneficial medications for syndrome progression and muscle loss in geriatrics, as has already been done in other medical fields [18]. Due to the infrequent assessment and diagnosis of sarcopenia, recruitment of patients of eligible cohorts through traditional sources remains a challenge. Consequently, past studies have struggled to reach the recruitment goals [19, 20]. While registries do not replace randomized controlled trials (RCT), they can help identify subgroups, questions and hypotheses for further examination in RCTs [21, 22]. A potential avenue for future clinical practice, therefore, involves RCTs based on registries [18, 22]. By utilizing the frequent hospitalizations of geriatric patients, data collection is easily carried out during their stays. As the registry currently includes over 400 datasets and continues to grow daily, we anticipate that further recruitment will lead to an even larger dataset, ultimately helping to mitigate the naturally high drop-out rates among advanced-aged patients.

In 2018 Sanchez-Rodriguez et al. finally proposed the establishment of an international registry on sarcopenia to provide a long-term source of data and patient cohort [23]. This initiative was not successful. The reasons are not published. They may have stemmed from various factors, such as potentially low participation or a data collection design that no longer aligned with the changed European data protection laws.

Additionally, technical difficulties may have contributed to the lack of success; therefore, our registry is also equipped with adjustments in this area. Our registry has a user-friendly interface and can be easily navigated. After instruction, researchers from different medical backgrounds can generate datasets. Accessible remotely, it enables a wide range of users to engage anytime at any place, while following the new European data protection laws [24]. This benefits future cooperation on a national and international basis and lines up with the launch of several cross-border initiatives that have recently been initiated in the EU region [25, 26]. The modular structure of our registry is in this collaboration context also advantageous. Future upcoming cooperation partners can incorporate their data and adapt the structure and finally, comprehensive research initiatives would also be highly welcomed by those affected. The results of a pilot study in the UK exploring the establishment of a patient registry for sarcopenia revealed a significant patient interest in further research participation. Remarkably, 98% of the recruited pilot participants expressed their willingness to be recruited for future studies [20].

### Limitations of registry-based research

Incomplete and invalid data are undoubtedly among the most significant challenges in every patient registry [18, 27]. Variability in input from different individuals at different times can compromise data quality, potentially leading to distortions and erroneous conclusions. To mitigate these concerns, we conduct regular quality control assessments involving all stakeholders [27]. Another possible limitation is the recruitment of patients during their hospital stay. Although this eases the recruitment of participants, it also poses a challenge to data quality, as acute illness, multiple different chronic diseases and exacerbation of comorbidities might influence their status at the time of assessment. No patient is in a “normal” state and this might potentially distort the accuracy and representativeness of the results. Next to this, the absence of fixed follow-up time points might also be a limitation. This leads to considerable variability in the assessment intervals (Fig. 1) and again, given the advanced age of the patients, the risk of drop-out due to patient death is also very high.

Another inherent disadvantage of our recruitment approach during hospital stays is also that we are unable to capture the time when the patient is not present. This prevents us from capturing potential factors that may influence subsequent assessments, such as changes during rehabilitation stays or social events in the patient’s life. Nonetheless, we can provide real-world data, in which certain variables and inconsistencies naturally affect outcomes or can create bias and errors in measurements [18]. The current single-center design can also be counted as a minor drawback but the integration of new collaborative partners in the future can address this issue.

## Conclusion

Despite the high prevalence and significant burden of sarcopenia, awareness for diagnosis and treatment in daily practice is low; efforts such as the consensus definition and the unified ICD-10 coding represent initial approaches to close this gap.

Targeted research with and for older patients is urgently needed to provide important insights and possible new therapeutic approaches.

The Munich Sarcopenia Registry (MUSAR) serves as an excellent example, offering a structured yet flexible data entry system that facilitates the creation of longitudinal datasets and addresses future research questions.

## Supplementary Information

Overview of assessments, questionnaires and measurements in the MUSAR registry

## Data Availability

The datasets used and analyzed in the current study are available from the corresponding author on request.
