# Supplementary material for: The MUnich SArcopenia Registry (MUSAR): Paving the way for increased visibility, more frequent diagnoses and innovative new treatment
Source: Z Gerontol Geriatr. 2025 Apr 8;58(3):197–202. doi: 10.1007/s00391-025-02428-2 (PMC12048408; doi:10.1007/s00391-025-02428-2)
Supplement: Supplementary file 1 — Overview of assessments, questionnaires and measurements in the MUSAR registry [file 391_2025_2428_MOESM1_ESM.docx]

**Appendix:**

**1. Measurements and assessments**

| **Table 1.** Overview of assessments and questionnaires in the MUSAR registry. |
| --- |
| **Modular assessment structure** |
| **Basis assessment** |
| Anthropometric measurements; Routine laboratory results; |
| Sociodemographic and socioeconomic background;  Medical history and Charlson Comorbidity Index (CCI) (1) |
| **Sarcopenia, frailty and osteoporosis assessment** |
| Strength, Assistance with walking, Rise from a chair, Climb stairs and Falls -Questionnaire (SARC-F) (2);  Skeletal muscle mass index (SMI) (3) |
| Fried`s phenotype Frailty Score (4) |
| Osteoporosis risk factor assessment and Dual X-ray Absorptiometry (DXA) (5) |
| **Geriatric assessment** |
| Hand grip strength (6); Chair rise test (7); Gait speed test (8); Timed-Up-and-Go (TUG) (9); Short-Physical Performance Battery (SPPB) (10); 400-Meter Walk Test 400MWT (11) |
| Mini Nutritional Assessment short form (MNA-SF) (12); |
| European Quality of Life Questionnaire (EQ5D) (13); Sarcopenia Quality of Life questionnaire (SarQoL) (14), Beck’s Depression Scale II (BDI II) (15); |
| Mini-Mental-Status-Examination (MMSE) (16); |
| Activity of daily living (ADL; Barthel Index) (17); Instrumental Activity of Daily Living (IADL) (18)**;** Falls Efficacy Scale International (FES-I) (19); |
| Assessment of oral health with dental status (20). |

**1.1. Basis Assessment**

Clinical and demographic data are collected at every assessment. This includes family history of relevant diseases, social background, and further medical history. Next to this, anthropometric measurements (e.g., height, weight, waist and hip circumference) are measured at each visit. The Charlson Comorbidity Index (CCI) is individually calculated (21). The numbers of diagnoses written in any medical report are the basis for index calculation, checked by prescribed medications. Additionally, the basis assessment captures common risk factors for sarcopenia like impairment of vision, hearing, sensing, vertigo/dizziness, pain, assessment of polyneuropathy, sleeping schedule and disorders, evaluation of depression through Beck`s depression scale, current number and type of medication, and further comorbidities (22).

The frequency of falls and fractures is recorded at each visit. Conclusively to encompass even more modifiable risk factors and comorbidities, our basic assessment is augmented by the inclusion of an extensive routine and endocrinologic blood laboratory.

**1.2 Sarcopenia, frailty, and osteoporosis assessment**

The main part of our registry assessment is geared towards sarcopenia. Additionally, explicit inquiries about frailty and osteoporosis are included, recognizing the substantial clinical overlaps and high frequency of co-occurrence among these conditions (23).

Screening by SARC-F questionnaire

The SARC-F questionnaire includes 5 components: strength, assistance walking, rising from a chair, climbing stairs, and falls. The scale score ranges from 0 to 10 (0-2 points to each component; 0 best to 10 worst). Scores from 0 to 3 represent healthy status and ≥ 4 represent positive screening for sarcopenia (2). The SARC-F has been translated, adapted, and validated into a German version in our clinic in cooperation with Nuremberg (24).

In the outpatient clinic, the questionnaire is used with self-completion. In the inpatient clinic, it is used as an interview-based questionnaire, inquired on average between the 3rd and 6th day after admission to exclude delirium and first acute symptoms affecting the answers.

Body composition by dual-energy X-ray absorptiometry (DXA)

The appendicular skeletal muscle is obtained as the sum of appendicular lean mass (ALM) of both arms and legs. The skeletal muscle mass index (SMI) is calculated by dividing the ALM by height in meters squared [kg/m²]. Cut-offs for SMI are set for men by <7.0 kg/m^2^ and for women by <5.5 kg/m^2^ (25).

Frailty status

Fried’s phenotype frailty criteria include three self-report questions (weight loss, exhaustion and low physical activity) and two performance-based measures [slowness (gait speed) and weakness (handgrip strength)] (4).

Osteoporosis assessment

All patients fill in an osteoporosis risk questionnaire covering different items such as family history, medical history, nutritional intake, falls and social participation (26). A dual X-ray Absorptiometry (DXA) of the lumbar spine and the proximal femur is performed (27). Bone mineral density is measured by DXA and in addition X-ray of the thoracic and lumbar spine is done in all patients with indication as described before [33]: The bone mineral density (BMD) of the lumbar spine is analyzed in line with the official guidelines of the International Society for Clinical Densitometry (ISCD), excluding vertebrae with abnormality or differences to adjacent vertebrae. Vertebral fractures are graduated according to the Genant classification.

All reported prior fractures are confirmed by X-ray or patient records.

**1.3. Geriatric Assessment**

The geriatric assessment is a comprehensive evaluation specifically tailored for aged patients that encompasses their functional capabilities and needs by using state-of-the-art functional tests and questionnaires (28).

Handgrip strength

Handgrip strength is measured using a hydraulic hand-held dynamometer (JAMAR, Los Angeles, CA). Three measurements are taken from each side, and the highest value of each side is used for diagnosis and taken for analysis.

Values <16 kg for women and <27 kg for men are considered positive for decreased hand grip strength (29).

Chair rise test

The chair rise test measures the time [in seconds (s)] a patient takes to get up five times from a sitting position. During the test, it is required to fold the arms in front of the chest to only assess the patient`s leg power. If the limit of 15 s for both sexes is exceeded, it is considered impaired (7).

Gait speed

To determine the gait speed, time is taken for a walking distance of 4 meters. The faster speed of two trials is used for the calculation of the 4 m speed in seconds per meter [s/m]. According to EWGSOP 2 criteria, a value of ≤0.8 m/s defines sarcopenia as severe (30).

Timed up and go (TUG)

The time-up and go (TUG) test is performed as described before (31): Time is measured for getting up from on a chair seated position, to get up, to walk to a line three meters away, to turn 180 degrees, and to sit down again. Cut off for limitation and severity are defined for patients who need ≥20 seconds(32).

The Short Physical Performance Battery (SPPB)

4 m gait speed, chair rise test in combination with balance test are used to calculate the SPPB score. A score (scale: 0–4) is assigned to the performance of each test. Individuals receive a score of 0 for each task they are unable to complete. Participants coded in the “unable to perform” category included those (a) who tried but were unable and (b) for whom the interviewer or participant felt it was unsafe. Summing the three individual categorical scores, a summary performance score is created for each participant (range: 0–12), with higher scores indicating better lower body function. In the walking test, the patients can use a cane, a walker, or other walking aid but no aid of another person (33).

The 400-Meter Walk Test (400MWT)

The 400-meter walk test (400MWT) is performed as described before(11). The time the patient takes to walk 400 meters (10 times 40m laps) is measured. The use of a cane or a walker is permitted. Breaks while standing, but not sitting, are also permitted. If the patient takes longer than 15 minutes, the test is stopped.

**1.4. Further Questionnaires**

MNA

The short version of the MNA is used to assess the nutritional status of the patients. Patients answered questions about food intake (0-2 points), weight loss (0-3 points), mobility (0-2 points), acute illnesses and mental stress (0-2 points) and neuropsychological problems (0-2 points). In addition, the BMI (0-3 points) is included in the calculation. Between 0 and 14 points can be achieved.

Values of 14-12 are considered normal nutritional status, 11-8 points as a risk of malnutrition, and 7-0 points as malnutrition (12).

QoL Questionnaire

Generic HR-QoL is measured using EuroQoL (EQ5D). This provides a profile of self-reported problems in five dimensions, health utility and a visual analog global self-rated health scale. For the EQ5D visual analog scale (EQ5D-VAS), each patient subjectively assesses his current health status on a printed scale of 0-100. A value of 100 represents the state of absolute health and a value of 0 represents the state of absolute illness. The EQ5D-VAS is included in the current analysis (34).

Also SarQoL® as a disease-specific questionnaire is included for further studies (35).

Dental status

The patients undergo a comprehensive dental assessment by the dental clinic of the LMU University Hospital Munich, Germany, including bite force analysis, plaque index evaluation and examination of denture fit and comfort.

**2. References**

1. Charlson ME, Pompei P, Ales KL, MacKenzie CR. A new method of classifying prognostic comorbidity in longitudinal studies: development and validation. J Chronic Dis. 1987;40(5):373–83.

2. Malmstrom TK, Morley JE. SARC-F: a simple questionnaire to rapidly diagnose sarcopenia. J Am Med Dir Assoc. 2013;14(8):531–2.

3. Tosato M, Marzetti E, Cesari M, Savera G, Miller RR, Bernabei R, et al. Measurement of muscle mass in sarcopenia: from imaging to biochemical markers. Aging Clin Exp Res. 2017 Feb 1;29(1):19–27.

4. Fried LP, Tangen CM, Walston J, Newman AB, Hirsch C, Gottdiener J, et al. Frailty in older adults: evidence for a phenotype. J Gerontol A Biol Sci Med Sci. 2001;56(3).

5. Drey M, Henkel M, Petermeise S, Weiß S, Ferrari U, Rottenkolber M, et al. Assessment of Bone and Muscle Measurements by Peripheral Quantitative Computed Tomography in Geriatric Patients. J Clin Densitom [Internet]. 2020 Oct 1 [cited 2023 Aug 31];23(4):604–10. Available from: https://pubmed.ncbi.nlm.nih.gov/30425007/

6. Schaupp A, Martini S, Schmidmaier R, Drey M. Diagnostic and therapeutic approach to sarcopenia. Z Gerontol Geriatr. 2021 Nov 1;54(7):717–24.

7. Hardy R, Cooper R, Shah I, Harridge S, Guralnik J, Kuh D. Is chair rise performance a useful measure of leg power? Aging Clin Exp Res. 2010;22(5–6):412.

8. Mehmet H, Robinson SR, Yang AWH. Assessment of Gait Speed in Older Adults. J Geriatr Phys Ther. 2020 Jan 1;43(1):42–52.

9. Bischoff HA, Stähelin HB, Monsch AU, Iversen MD, Weyh A, von Dechend M, et al. Identifying a cut-off point for normal mobility: a comparison of the timed “up and go” test in community-dwelling and institutionalised elderly women. Age Ageing. 2003 May;32(3):315–20.

10. Lauretani F, Ticinesi A, Gionti L, Prati B, Nouvenne A, Tana C, et al. Short-Physical Performance Battery (SPPB) score is associated with falls in older outpatients. Aging Clin Exp Res. 2019 Oct 1;31(10):1435–42.

11. Vestergaard S, Patel K V., Bandinelli S, Ferrucci L, Guralnik JM. Characteristics of 400-Meter Walk Test Performance and Subsequent Mortality in Older Adults. Rejuvenation Res. 2009 Jun;12(3):177–84.

12. Kaiser MJ, Bauer JM, Ramsch C, Uter W, Guigoz Y, Cederholm T, et al. Validation of the Mini Nutritional Assessment short-form (MNA-SF): a practical tool for identification of nutritional status. J Nutr Health Aging. 2009;13(9):782–8.

13. Rabin R, Charro F de. EQ-SD: a measure of health status from the EuroQol Group. Ann Med. 2001 Jan;33(5):337–43.

14. Beaudart C, Biver E, Reginster JY, Rizzoli R, Rolland Y, Bautmans I, et al. Validation of the SarQoL®, a specific health-related quality of life questionnaire for Sarcopenia. J Cachexia Sarcopenia Muscle. 2017;8(2):238–44.

15. Beck AT, Steer RA, Ball R, Ranieri W. Comparison of Beck Depression Inventories -IA and -II in psychiatric outpatients. J Pers Assess. 1996 Dec;67(3):588–97.

16. Fujii M, Butler JP, Hirazakura A, Sasaki H. Mini-Emotional State Examination for dementia patients. Geriatr Gerontol Int. 2014 Apr 1;14(2):508–13.

17. MAHONEY FI, BARTHEL DW. FUNCTIONAL EVALUATION: THE BARTHEL INDEX. Md State Med J. 1965 Feb;14:61–5.

18. Iwamura M, Kanauchi M. A cross-sectional study of the association between dynapenia and higher-level functional capacity in daily living in community-dwelling older adults in Japan. BMC Geriatr. 2017;17(1):1–6.

19. Figueiredo D, Neves M. Falls Efficacy Scale-International: Exploring psychometric properties with adult day care users. Arch Gerontol Geriatr. 79:145–50.

20. Razak PA, Richard KMJ, Thankachan RP, Hafiz KAA, Kumar KN, Sameer KM. Geriatric oral health: a review article. J Int Oral Health. 2014;6(6):110–6.

21. Charlson ME, Pompei P, Ales KL, MacKenzie CR. A new method of classifying prognostic comorbidity in longitudinal studies: development and validation. J Chronic Dis. 1987;40(5):373–83.

22. Yuan S, Larsson SC. Epidemiology of sarcopenia: Prevalence, risk factors, and consequences. Vol. 144, Metabolism: Clinical and Experimental. Metabolism; 2023.

23. Greco EA, Pietschmann P, Migliaccio S. Osteoporosis and sarcopenia increase frailty syndrome in the elderly. Front Endocrinol (Lausanne). 2019;10(APR):255.

24. Drey M, Ferrari U, Schraml M, Kemmler W, Schoene D, Franke A, et al. German Version of SARC-F: Translation, Adaption, and Validation. J Am Med Dir Assoc. 2020 Jun 1;21(6):747-751.e1.

25. Gould H, Brennan SL, Kotowicz MA, Nicholson GC, Pasco JA. Total and Appendicular Lean Mass Reference Ranges for Australian Men and Women: The Geelong Osteoporosis Study. Calcif Tissue Int. 2014 Apr;94(4):363–72.

26. Neuerburg C, Mittlmeier L, Schmidmaier R, Kammerlander C, Böcker W, Mutschler W, et al. Investigation and management of osteoporosis in aged trauma patients: a treatment algorithm adapted to the German guidelines for osteoporosis. J Orthop Surg Res. 2017 Jun 8;12(1).

27. Drey M, Henkel M, Petermeise S, Weiß S, Ferrari U, Rottenkolber M, et al. Assessment of Bone and Muscle Measurements by Peripheral Quantitative Computed Tomography in Geriatric Patients. J Clin Densitom. 2020 Oct 1;23(4):604–10.

28. Schippinger W. Comprehensive geriatric assessment. Wien Med Wochenschr. 2022 Apr 1;172(5–6):122–5.

29. Cruz-Jentoft AJ, Bahat G, Bauer J, Boirie Y, Bruyère O, Cederholm T, et al. Sarcopenia: revised European consensus on definition and diagnosis. Age Ageing. 2019 Jan;48(1):16–31.

30. Cruz-Jentoft AJ, Bahat G, Bauer J, Boirie Y, Bruyère O, Cederholm T, et al. Sarcopenia: revised European consensus on definition and diagnosis. Age Ageing. 2019 Jan;48(1):16–31.

31. Bischoff HA, Stähelin HB, Monsch AU, Iversen MD, Weyh A, von Dechend M, et al. Identifying a cut-off point for normal mobility: a comparison of the timed “up and go” test in community-dwelling and institutionalised elderly women. Age Ageing. 2003;32(3):315–20.

32. Bischoff HA, Stähelin HB, Monsch AU, Iversen MD, Weyh A, von Dechend M, et al. Identifying a cut-off point for normal mobility: a comparison of the timed “up and go” test in community-dwelling and institutionalised elderly women. Age Ageing [Internet]. 2003 May [cited 2023 Aug 31];32(3):315–20. Available from: https://pubmed.ncbi.nlm.nih.gov/12720619/

33. Lauretani F, Ticinesi A, Gionti L, Prati B, Nouvenne A, Tana C, et al. Short-Physical Performance Battery (SPPB) score is associated with falls in older outpatients. Aging Clin Exp Res [Internet]. 2019 Oct 1 [cited 2023 Aug 31];31(10):1435–42. Available from: https://link.springer.com/article/10.1007/s40520-018-1082-y

34. Rabin R, Charro F de. EQ-SD: a measure of health status from the EuroQol Group. Ann Med. 2001 Jan;33(5):337–43.

35. Beaudart C, Biver E, Reginster JY, Rizzoli R, Rolland Y, Bautmans I, et al. Validation of the SarQoL®, a specific health-related quality of life questionnaire for Sarcopenia. J Cachexia Sarcopenia Muscle. 2017;8(2):238–44.
